# Dosimetric analysis of tangent-based volumetric modulated arc therapy with deep inspiration breath-hold technique for left breast cancer patients

**DOI:** 10.1186/s13014-018-1170-3

**Published:** 2018-11-26

**Authors:** Pei-Chieh Yu, Ching-Jung Wu, Yu-Lun Tsai, Suzun Shaw, Shih-Yu Sung, Louis Tak Lui, Hsin-Hua Nien

**Affiliations:** 1 Department of Radiation Oncology, Cathay General Hospital, Taipei, Taiwan; 2 School of Medicine, China Medical University, Taichung, Taiwan; 3 Department of Radiation Oncology, Oncology Treatment Center, Sijhih Cathay General Hospital, New Taipei City, Taiwan; 4 Department of Radiation Oncology, National Defense Medical Center, Taipei, Taiwan; 5 Department of Biomedical Engineering, I-Shou University, Kaohsiung, Taiwan; 6 School of Medicine, Fu Jen Catholic University, New Taipei City, Taiwan

**Keywords:** Tangent-based volumetric modulated arc therapy, Breast cancer, Tangent-based intensity modulated radiation therapy, Deep inspiration breath-hold

## Abstract

**Background:**

Tangent-based intensity modulated radiation therapy (TIMRT) is a common adjuvant radiotherapy strategy for breast cancer patients. This study compared the dosimetric characteristics of tangent-based volumetric modulated arc therapy (TVMAT) and TIMRT for left breast cancer patients during deep inspiration breath-hold (DIBH) and free breathing (FB) techniques.

**Methods:**

Fourteen patients with left breast cancer after breast-conserving surgery were included. The first arc started at 331.8–353.6 degrees and stopped at 281.8–315.0 degrees. The third arc started at 123.2–149.1 degrees and stopped at 88.0–96.0 degrees. The second and fourth arcs were reverse arcs of first and third arcs. DIBH-TIMRT inversing plans were generated using opposing tangential fields. Wilcoxon signed rank test and Spearman correlation were used to examine the significance of dose difference.

**Results:**

Compared with FB-TVMAT, the mean heart dose of DIBH-TVMAT plans was reduced from 7.9 Gy to 3.2 Gy (*p* < 0.001). The average left lung volume receiving 30 Gy or more (V30Gy) was reduced from 12.9 to 5.7% (*p* < 0.001). DIBH-TVAMT plans resulted in a lower mean dose to the contralateral breast and lung (2 Gy and 0.7 Gy vs 3.4 Gy and 1.5 Gy, respectively) as compared to FB-TVMAT plans. Compared with DIBH-TIMRT, the average left lung V30Gy of DIBH-TVMAT plans was reduced from 8.5 to 5.7% (*p* = 0.031). As for low-dose areas, exposure of the left lung, right breast, heart and right lung volume with 10 Gy or more was not significantly different between the IMRT- and VMAT-plans.

**Conclusions:**

DIBH-TVMAT for left breast cancer treatment retains treatment plan quality similar to the DIBH-IMRT technique without compromising dose restrictions to the heart, right breast and right lung. DIBH-TVMAT increased left lung protection but still had higher V5Gy to right breast and substantially higher V5Gy to heart. For left breast cancer patients receiving treatment with the DIBH technique, DIBH-TVMAT provides better treatment quality and is a safe and feasible treatment strategy.

## Background

For patients with breast cancer receiving breast conserving surgery, tangential beam arrangement is a traditional technique for adjuvant radiation therapy planning. Compared with tangential beam arrangement technique, tangent-based intensity modulated radiation therapy (TIMRT) has been studied in recent years and showed advantages in target coverage conformity and homogeneity. TIMRT has become a common adjuvant radiotherapy strategy for breast cancer patients [1–4].

Beside treatment planning technique, other supporting techniques such as deep inspiration breathing hold technique (DIBH), respiration gating, and 3D body surface measurement were investigated to improve treatment quality. Several studies have suggested that the DIBH technique diminishes undesired radiation exposure to surrounding normal tissue, including lung and heart tissues [5–8]. The benefits of DIBH were especially emphasized on heart dose reduction for left breast cancer patients receiving chest wall irradiation [9–11]. A large retrospective cohort trial by Darby et al. revealed the association between incidental exposure of the heart to radiotherapy for breast cancer and increase subsequent rate of ischemic heart disease [12]. DIBH-TIMRT provided a better protective treatment strategy, especially for left breast cancer patients [1, 2].

Along with the evolution of radiotherapy machines and treatment planning systems, the advanced planning technique of volumetric modulated arc therapy (VMAT) was proved to have the general benefits of target coverage conformity, homogeneity, organ at risk (OAR) sparing, and delivery time reduction compared to intensity modulated radiation therapy (IMRT) [13–16]. However, instead of reducing the high-dose regions of surrounding OARs, the low-dose area of VMAT represents a treatment planning challenge when the organs at risk (lungs and heart) are very close to the planning target volume (PTV; i.e., left breast). It is also a concern that radiation exposure may lead to a second malignancy or unexpected heart disease for patients with long-term survival. Viren et al.’s study showed that continuous VMAT (cVMAT) increased the mean dose of contralateral breast significantly [17]. Thus, VMAT may not serve as first choice technique for breast cancer.

To avoid the disadvantage of VMAT, tangent-based VMAT (TVMAT) technique was used for treatment planning in our institution. This study was conducted to compare dosimetric characteristics of DIBH-TVMAT and DIBH-TIMRT of left breast cancer patients. Dosimetric data of the right lung, right breast tissue, heart and left lung comparing DIBH-TVMAT, free-breathing (FB)-TVMAT, and DIBH-TIMRT were calculated.

## Materials and methods

This study was approved by the institutional review board as CGH-P106079.

### Patient selection, simulation, and target volume delineation

Our patient database was reviewed and patients were identified as meeting the following criteria: (1) patients with left breast cancer after receiving breast-conserving surgery and adjuvant radiotherapy between Sept. 1, 2013 and Sept. 1, 2014, (2) patients who received both DIBH and FB simulation CT scans, and (3) patients who received breast irradiation only. Exclusion criteria were: (1) patients who received regional nodal irradiation (2) patients who received previous chest wall surgery other than breast conserving surgery, (3) patients who received previous breast irradiation or chest irradiation. Fourteen patients with left breast cancer after breast-conserving surgery were included in this study. The mean age of patients was 47 ± 8 years.

Patients were positioned supine with arms raised above the head on a vacuum bag. Computed tomography (CT) simulation was performed with intravenous contrast. CT slices were acquired with a 16-slice CT scanner (Discovery CT590 RT, General Electric (GE) Healthcare, Waukesha, WI). CT acquisition was with 2.5 mm slice thickness extending from the top of the second cervical vertebral body to the bottom of the fourth lumbar vertebral body.

Each patient underwent DIBH and FB CT scans. Breath-hold level was recorded by infrared reflecting marker in the anteroposterior direction by Real-time Position Management system (Varian Medical System, Palo Alto, USA) during the CT procedure.

Clinical target volume (CTV) was contoured by the same radiation oncologist for each patient. CTV only included left breast residual tissue. Regional nodal area was not included in CTV. The PTV was defined as a three-dimensional expansion of the CTV with a 5.0-mm margin in all directions. PTV was cropped 3 mm from the skin. For each scan, a TVMAT plan was designed. In addition, a TIMRT plan was designed using a DIBH CT scan. The average PTV volume of DIBH was 525.1 ± 194 cm^3^ and 502.4 ± 182.2 cm^3^ for FB.

### Treatment planning

**Fig. 1:**
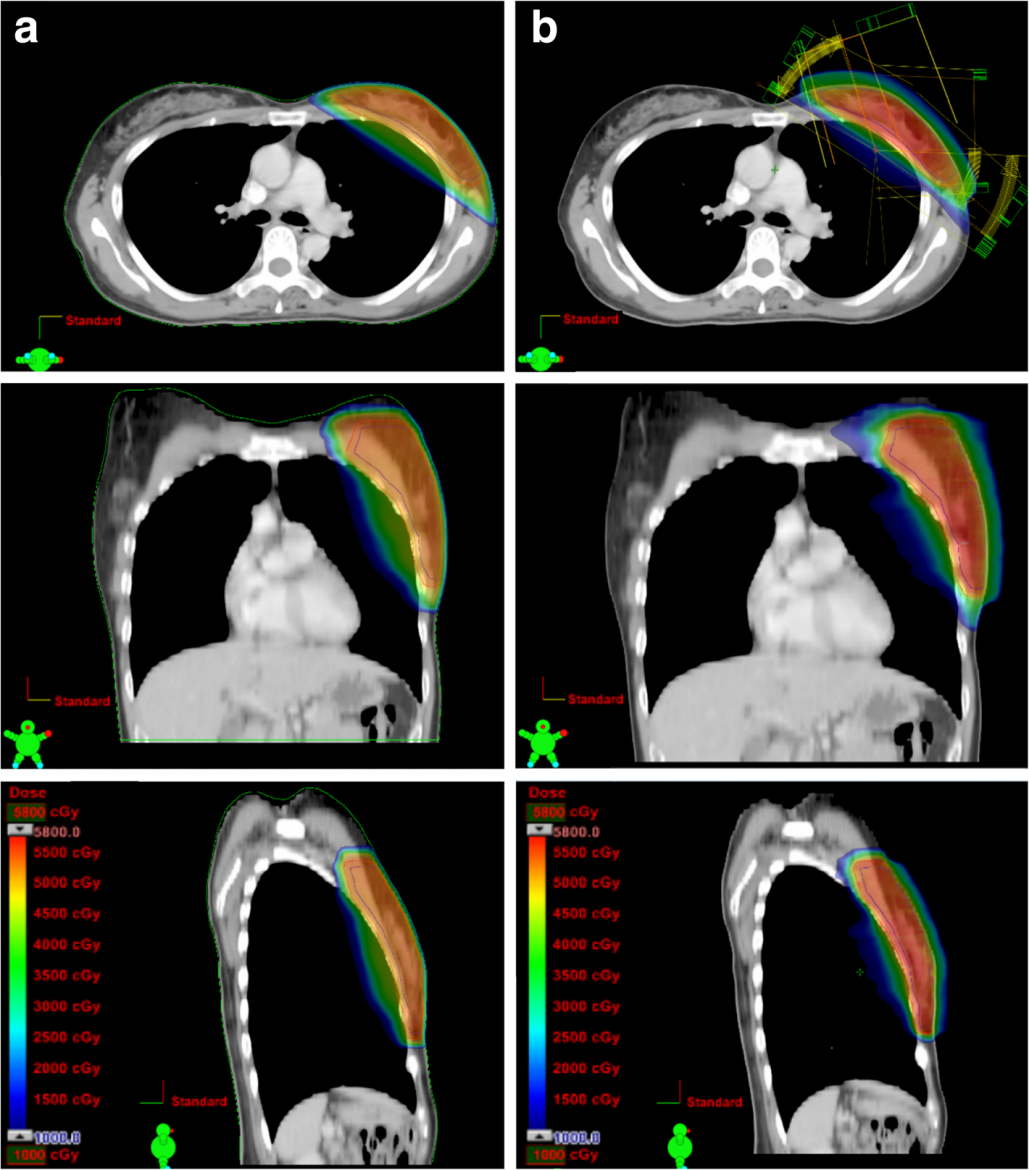
Dose distribution and beam arrangement with axial, coronal and sagittal views with each technique. Dose distribution and beam arrangement on axial, coronal and sagittal views of one patient with (**a**) tangent-based intensity modulated radiation therapy (TIMRT) during deep inspiration breath-hold (DIBH); (**b**) tangent-based volumetric modulated arc therapy (TVMAT) during deep inspiration breath-hold (DIBH) technique

DIBH-TVMAT plans were generated using mono-isocenteric technique with four partial rotation arcs for left breast residual tissue. The first arc started at 331.8–353.6 degrees and stopped at 281.8–315.0 degrees. The third arc started at 123.2–149.1 degrees and stopped at 88.0–96.0 degrees. The second and fourth arcs were reverse arcs of first and third arcs, respectively (Fig. 1). Gantry setting of FB-TVMAT plan was the same as DIBH-TVMAT plan for each patient. DIBH-TIMRT plans were generated using opposing tangential fields encompassing the whole breast tissue. In TVMAT plan, virtual bolus was added to account for small changes in size and position of the target, and possible edema according to Giorgia et al. study [18]. All plans were done by same physicist.

Only whole breast irradiation plans were included for plan comparison in this study. The total dose prescribed was 50.0 Gy, with 2.0 Gy per fraction per day. The aim of treatment planning was to achieve at least 95% of the volume of PTV receiving 47.5 Gy (95% of 50.0 Gy) and the left lung volume receiving 20 Gy or more (V20Gy) ≦ 10% while keeping the right lung below a mean dose of 5 Gy. The dose constrains and relative priority for target volumes and organs at risk are listed in Table 1. For each patient, DIBH-TVMAT was planned first. Gantry setting of FB-TVMAT was the same as DIBH-TVMAT plan. First aim for FB-TVMAT plan was to achieve volume receiving 95% of prescribed dose (V95%) of PTV ≥ 95%. Then second aim was to decrease OAR dose as much as possible. The aim of treatment plan optimization was to decrease OAR dose as low as possible under the precondition of V95% of PTV ≥ 95%. Once the dose evaluation showed V95% of PTV < 95%, the optimization process was terminated and the plan was set as final plan. Three treatment techniques with similar PTV conformity and homogeneity were planned for every patient.

**Table 1:** Dose constrains and relative priority for target volumes and organs at risk

Structures	Dose constrains	Priority
PTV	Dmin > 47.5 Gy	600
	Dmax < 55 Gy	800
Heart	V30Gy (%) < 1%	200
	V10Gy (%) < 20%	200
	V5Gy (%) < 40%	200
Left lung	V20Gy (%) < 10%	500
	V10Gy (%) < 30%	500
	V5Gy (%) < 40%	500
Right lung	Dmean < 5 Gy	200
	D2% (Gy) < 10 Gy	200
Right breast	Dmean < 5 Gy	400
	D2% (Gy) < 10 Gy	400

Legend: Dose objectives and relative priority were used for planning optimization

Radiation treatment was planned with 6 MV photon energy using the Eclipse treatment planning system Version 11 (Varian Medical System, Palo Alto, USA). The grid size utilized for dose calculation was 2.5 mm. A maximum dosage rate of 600 monitor units per minute (MU/min) was used. The Anisotropic Analytical Algorithm (Version 11.0.31) was performed for volume dose calculation. Progressive Resolution Optimizer (Version 11.0.31) was used for VMAT optimization.

### Data analysis

Wilcoxon signed rank test was used to examine the significance of dose difference among different treatment plans. Statistical significance was set at *p* < 0.05. Spearman correlation was used to examine the correlation significance between dose and dose reduction. The box plot showed the data range for dosimetric parameter comparison.

## Results

### DIBH-TIMRT vs. DIBH-TVMAT

The dose distribution comparison of DIBH-TIMRT and DIBH-TVMAT technique on axial, coronal and sagittal views is shown in Fig. 1. DIBH-TVMAT had better conformity index (CI), and also better V95% of PTV than DIBH-TIMAT, but there was no significant PTV conformity or homogeneity difference between the DIBH-TIMRT group and DIBH-TVMAT group (Table 2). The average MU were 375.7 ± 40.6 for DIBH-TVMAT plans, which was significantly lower compared with 524.0 ± 192.3 for DIBH-TIMRT plans (*p* = 0.009; Table 2).

**Table 2:** Treatment plan evaluation result of DIBH-TVMAT vs. DIBH-TIMRT plans

		DIBH-TVMAT		DIBH-TIMRT		*p*-value
		Mean	SD	Mean	SD	
PTV	CI	0.94	0.05	0.92	0.03	0.120
	HI	0.17	0.02	0.16	0.01	0.074
PTV	V95% (%)	97.0	0.9	95.8	1.1	0.783
Monitor Units (MU)		375.7	40.6	524	192.3	0.009*

Legend: Treatment plan evaluation result shows MUs of radiation delivery, conformity index (CI), homogeneity index (HI) and volume receiving 95% of prescribed dose or more (V95%) of planning target volume (PTV) with DIBH-TVMAT technique and DIBH-TIMRT techniques

The average left lung volume receiving 30 Gy or more (V30Gy) was 5.7% ± 3.2% for DIBH-TVMAT, compared with 8.5% ± 3.3% for DIBH-TIMRT (*p* = 0.031). For high-dose region, DIBH-TVMAT plans had higher doses received by 2% (D2%) of right lung and higher D2% of right breast compared with DIBH-TIMRT plans (*p* = 0.04, 0.029, respectively). For low-dose areas, DIBH-TVMAT plans had higher volume receiving 5 Gy or more (V5Gy) of right breast compared with DIBH-TIMRT plans (*p* = 0.002). The results showed higher V5Gy of heart with DIBH-TVAMT plans compared with DIBH-TIMRT plans but didn’t reach statistical significance. The average left lung, right breast, heart and right lung volumes receiving 10 Gy or more (V10Gy) had no significant difference between two techniques (Table 3). There was no significant difference of heart dose among mean dose, D2%, V30Gy, or V10Gy between DIBH-TVMAT and DIBH-TIMAT. There was no significant difference of left anterior descending artery (LAD) dose among Dmax and dose received by 10% of volume (D10%) between two techniques. The results also revealed no significant difference in right lung dosage (including V5Gy and V10Gy) when comparing DIBH-TVMAT to DIBH-TIMRT (*p* = 0.943 for V5Gy, *p* = 0.447 for V10Gy).

**Table 3:** Dosimetric parameters of OARs with DIBH-TVMAT vs. DIBH-TIMRT

		DIBH-TVMAT		DIBH-TIMRT		*p*-value
		Mean	SD	Mean	SD	
Heart	mean dose (Gy)	3.2	2.1	1.9	1.8	0.088
	D2% (Gy)	12.7	10.0	10.6	11.3	0.610
	V30Gy (%)	0.4	1.3	0.5	1.2	0.880
	V10Gy (%)	4.8	7.0	2.2	3.9	0.252
	V5Gy (%)	19.4	17.8	7.7	14.4	0.066
LAD	Dmax (Gy)	20.1	15.3	20.6	17.7	0.934
	D10% (Gy)	17.1	14.0	17.3	16.9	0.978
Left Lung	mean dose (Gy)	6.6	1.9	7.0	2.3	0.565
	D2% (Gy)	40.1	7.5	44.3	3.2	0.064
	V30Gy (%)	5.7	3.2	8.5	3.3	0.031*
	V20Gy (%)	9.2	4.3	12.2	4.4	0.085
	V10Gy (%)	18.7	6.0	17.6	6.4	0.645
	V5Gy (%)	31.7	7.2	28.5	12.6	0.423
Right Lung	mean dose (Gy)	0.7	0.3	0.5	0.8	0.386
	D2% (Gy)	3.7	1.4	1.4	2.3	0.04*
	V10Gy (%)	0.0	0.1	0.1	0.3	0.447
	V5Gy (%)	1.0	1.0	0.9	3.2	0.943
Right Breast	mean dose (Gy)	2.0	0.6	3.1	8.9	0.661
	D2% (Gy)	7.8	2.7	4.2	5.1	0.029*
	V10Gy (%)	1.0	1.6	0.9	2.5	0.903
	V5Gy (%)	9.1	6.2	2.2	4.2	0.002*

Legend: Dosimetric parameters of OARs including heart, left lung, right lung, and breast with TVMAT and TIMRT under DIBH technique are presented

The left lung mean dose reduction from DIBH-TIMRT to DIBH-TVMAT correlated moderately with left lung mean dose of DIBH-TIMAT (left lung mean dose: Spearman’s *r* = 0.591; *p* = 0.025).

**Fig. 2:**
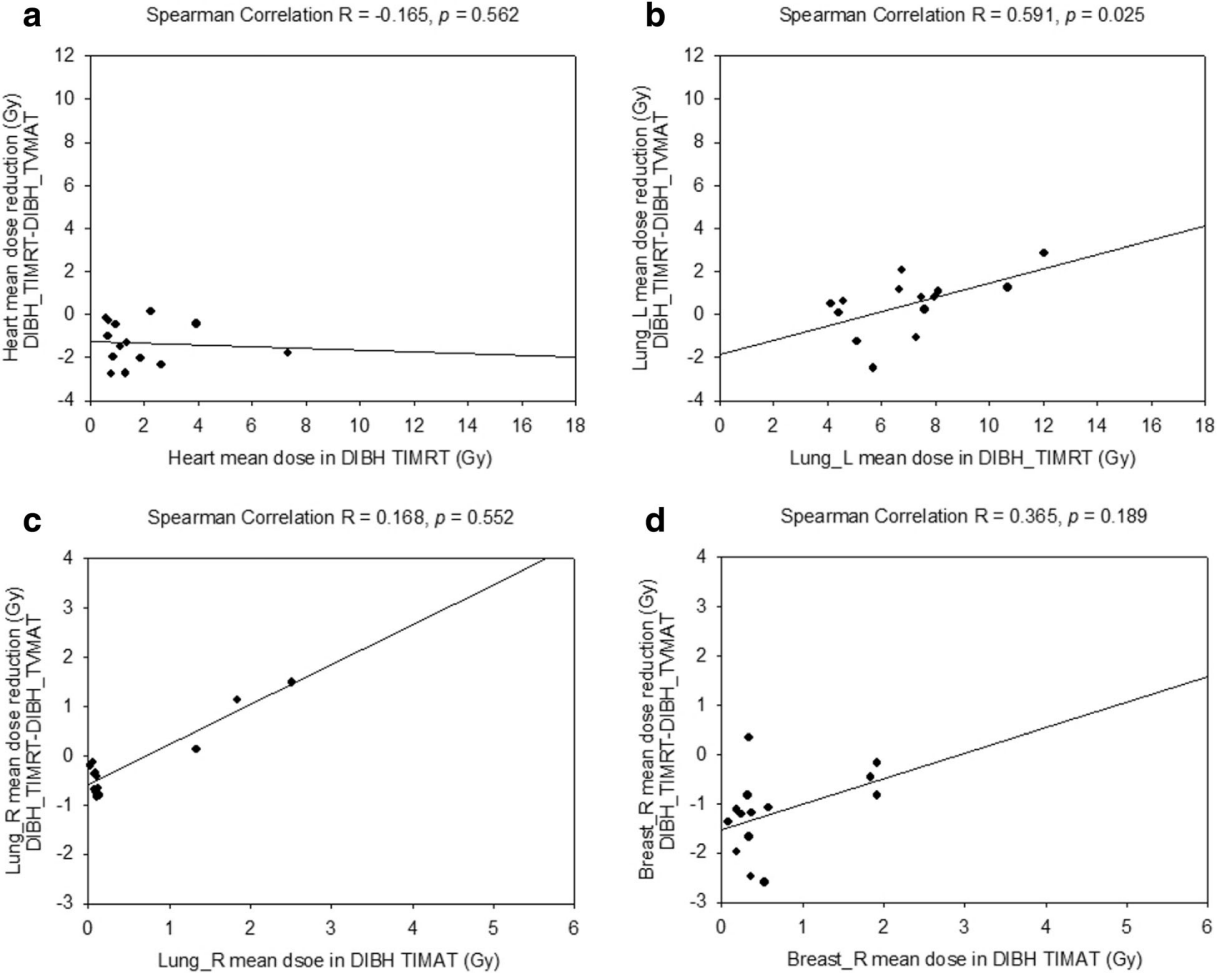
The correlation between reduced OAR dose with TVMAT and OAR dose with TIMRT under DIBH. There was no significant correlation between (**a**) mean heart dose reduction, (**b**) left lung (lung_L) mean dose reduction, (**c**) right lung (lung_R) mean dose reduction, or (**d**) right breast (breast_R) mean dose reduction using DIBH-TVMAT and each OAR’s mean dose of DIBH-TIMRT, respectively. The solid line represents linear regression fit for all 14 patients

The heart, right breast, and right lung mean dosage difference between DIBH-TIMAT and DIBH-TVMAT did not correlate well with DIBH-TIMAT mean dose (Fig. 2).

### DIBH-TVMAT vs. FB-TVMAT

The DIBH-TVMAT group experienced significantly reduced dosimetric parameters of heart, left lung, and right lung radiation compared with the FB-TVMAT group. Compared with FB-TVMAT, the average mean heart dose of DIBH-TVMAT plans was reduced from 7.9 ± 3.6 Gy to 3.2 ± 2.1 Gy (*p* < 0.001). DIBH-TVMAT had significantly lower Dmax of LAD compared with FB-TVMAT (20.1 ± 15.3 Gy vs 40.4 ± 12.3 Gy, *p* = 0.001). The D10% of LAD was reduced from 36.47 ± 13.3 Gy of FB-TVMAT to 17.1 ± 14.0 Gy of DIBH-TVMAT (*p* = 0.001). The average left lung V20Gy was reduced from 20.7% ± 4.7% of FB-TVMAT to 9.2% ± 4.3% of DIBH-TVMAT (*p* < 0.001). V20Gy of the left lung of all DIBH-TVMAT plans was lower than 17%. DIBH-TVMAT resulted in significantly lower V5Gy of left lung compared with FB-TVMAT (31.7 ± 7.2 Gy vs 53.6 ± 8.6 Gy, *p* < 0.001).

**Fig. 3:**
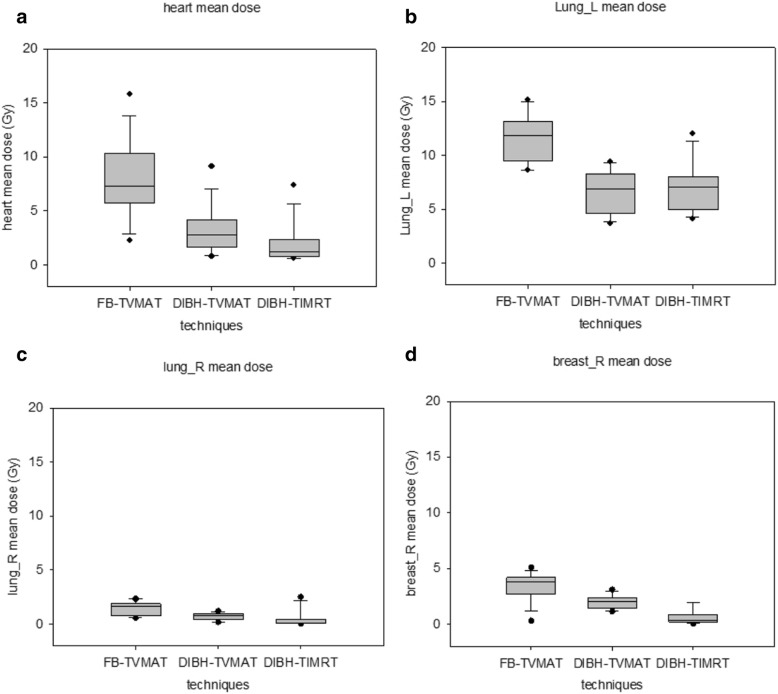
Comparison of mean doses of organs at risk (OAR) with each technique. Box plots for (**a**) mean heart dose, (**b**) left lung (lung_L) mean dose, (**c**) right lung (lung_R) mean dose, and (**d**) right breast (breast_R) mean dose. The boxes indicate 25th and 75th percentiles of dose distribution, while horizontal lines within the boxes indicated the median. Error bars show standard deviation for extreme values, and black dots denote maximum and minimum values

DIBH-TVMAT plans resulted in significantly lower mean dose of right breast than FB-TVMAT plans (2 ± 0.6 Gy vs 3.4 ± 1.3 Gy, *p* = 0.001). For right lung, DIBH-TVMAT plans resulted in significantly lower mean dose than FB-TVMAT plans (0.7 ± 0.3 Gy vs 1.5 ± 0.6 Gy, *p* < 0.001). The average V5Gy was reduced from 24.0% ± 11.3% of FB-TVMAT plans to 9.1% ± 6.2% of DIBH-TVMAT plans for the right breast (*p* < 0.001) and from 6.7% ± 4.9 to 1.0% ± 1.0% for the right lung (*p* < 0.001). The mean dose and V5Gy of all evaluated organs were reduced significantly by the DIBH-TVMAT technique (Fig. 3; Table 4).

**Table 4:** Dosimetric parameters of organ at risk with DIBH-TVMAT vs. FB-TVMAT

		DIBH-TVMAT		FB-TVMAT		*p*-value
		Mean	SD	Mean	SD	
Heart	mean dose (Gy)	3.2	2.1	7.9	3.6	< 0.001
	D2% (Gy)	12.7	10.0	31.5	12.3	< 0.001
	V30Gy (%)	0.4	1.3	3.6	4.5	0.017
	V10Gy (%)	4.8	7.0	25.5	14.0	< 0.001
	V5Gy (%)	19.4	17.8	51.5	22.4	< 0.001
LAD	Dmax (Gy)	20.1	15.3	40.4	12.3	0.001
	D10% (Gy)	17.1	15.0	36.5	13.3	0.001
Left Lung	mean dose (Gy)	6.6	1.9	11.8	2.1	< 0.001
	D2% (Gy)	40.1	7.5	48.4	1.8	< 0.001
	V30Gy (%)	5.7	3.2	12.9	3.7	< 0.001
	V20Gy (%)	9.2	4.3	20.7	4.7	< 0.001
	V10Gy (%)	18.7	6.0	37.6	7.2	< 0.001
	V5Gy (%)	31.7	7.2	53.6	8.6	< 0.001
Right Lung	mean dose (Gy)	0.7	0.3	1.5	0.6	< 0.001
	D2% (Gy)	3.7	1.4	6.8	2.2	< 0.001
	V10Gy (%)	0.0	0.1	0.5	0.5	0.002
	V5Gy (%)	1.0	1.0	6.7	4.9	< 0.001
Right Breast	mean dose (Gy)	2.0	0.6	3.4	1.3	0.001
	D2% (Gy)	7.8	2.7	9.2	3.3	0.232
	V10Gy (%)	1.0	1.6	2.5	2.8	0.115
	V5Gy (%)	9.1	6.2	24.0	11.3	< 0.001

Legend: Dosimetric parameters of organ at risk (OAR) including heart, left lung, right lung, and breast with tangent-based volumetric modulated arc therapy (TVMAT) under deep inspiration breath-hold (DIBH) technique and free breathing (FB)

**Fig. 4:**
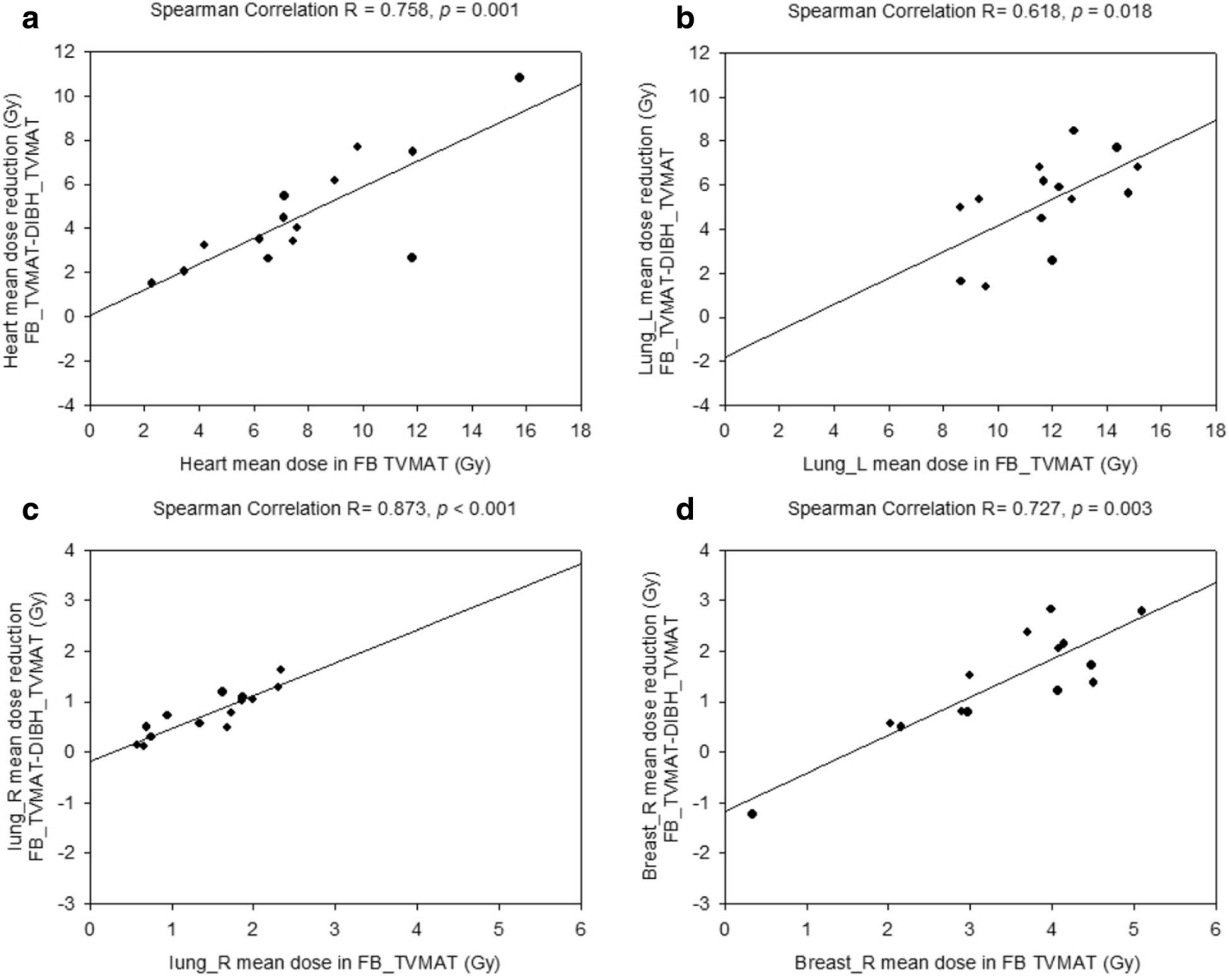
Correlation between reduced OAR dose with DIBH and OAR dose with FB under TVMAT. This figure shows the correlation between (**a**) mean heart dose reduction, (**b**) left lung (lung_L) mean dose reduction, (**c**) right lung (lung_R) mean dose reduction, (**d**) right breast (breast_R) mean dose reduction of DIBH-TVMAT and each OAR mean dose of FB-TVMAT, respectively. The solid line represents linear regression fit for all 14 patients

The mean dose reduction of OAR from FB-TVMAT to DIBH-TVMAT correlated strongly with each OAR mean dose of FB-TVMAT (heart mean dose: Spearman’s *r* = 0.758, p = 0.001; left lung mean dose: Spearman’s *r* = 0.618, *p* = 0.018; right lung mean dose: Spearman’s *r* = 0.873, p < 0.001; right breast mean dose: Spearman’s *r* = 0.727, *p* = 0.003; Fig. 4).

## Discussion

This study compared radiotherapy treatment planning with DIBH-TVMAT, DIBH-TIMRT, and FB-TVMAT techniques for left breast. To our knowledge, the herein presented data is among the first investigating studies with DIBH-TVMAT technique for breast irradiation. Higher D2% of the right lung and right breast were noted with DIBH-TVMAT compared with DIBH-TIMRT. Right breast V5Gy with DIBH-TVMAT is higher than that of DIBH-TIMRT. However, for V10Gy of the right breast and right lung, which was used for possible radiation pneumonitis evaluation, DIBH-TVMAT was as low as DIBH-TIMRT. For the most important organ, the heart, all parameters of DIBH-TVMAT achieved performance as good as DIBH-TIMRT. Furthermore, DIBH-TVMAT protected the left lung, with significantly lower V30Gy than DIBH-TIMRT. DIBH-TVMAT had a tendency toward a higher HI but there was no significant difference in HI and CI between DIBH-TVMAT and DIBH-TIMRT. For patients with larger chest wall curves, TVMAT contributed to higher heterogeneity of PTV dose coverage, which may lead to more adverse effects.

Jin et al. [19] showed that TIMRT for left breast cancer treatment reduced radiation dose exposure for normal tissues and maintained reasonable target homogeneity compared with conventional tangential wedge-based fields, field-in-field, multi-field IMRT, and VMAT techniques, and therefore VMAT was not recommended for left breast cancer radiotherapy. Viren et al. [17] showed that cVMAT for left breast cancer radiotherapy increased the mean dose of contralateral breast significantly, but dosages of the heart and left lung can be reduced without increasing the dose to the right breast or lung with tangential VMAT (tVMAT) technique under FB conditions. Pham et al. [2] investigated the potential heart sparing effect of cVMAT. Their study showed that cVMAT-DIBH significantly spared the heart volume received radiation 20 Gy and above. However, the study also found cVMAT-DIBH contributed to a significantly higher V5Gy of the heart, significantly higher V5Gy, V10Gy, and mean dose of right lung, and significantly higher V5Gy and mean dose of right breast compared with tangential intensity-modulated radiotherapy-DIBH. Instead of cVMAT, we used TVMAT technique for treatment planning. DIBH-TVMAT had better CI and V95% of PTV compared with DIBH-TIMRT. According to Darby et al., higher incidental radiotherapy exposure of the heart is associated with higher risk of major coronary events by 7.4% per gray of mean dose of the heart with no apparent threshold [12]. Our results showed that the DIBH-TVMAT technique significantly spared the heart, including mean dosage, D2%, V30Gy, V10Gy and V5Gy as well as DIBH-TIMAT. DIBH-TVMAT had higher D2% for high dose area and higher V5Gy for low dose area of the heart than DIBH-TIMRT but without statistical significance. TVMAT provided better heart protection compared to cVMAT. However, while cVMAT-DIBH had significant lower mean dose of LAD than tangential intensity-modulated radiotherapy-DIBH [2], our DIBH-TVMAT only achieved non-inferior protection of LAD than DIBH-TIMRT. DIBH-TVMAT spared heart as well as DIBH-TIMRT but risk of major coronary artery disease remains a concerned issue. For other OAR, there was no statistically significant mean dose difference among the left lung, right lung, or right breast between DIBH-TVMAT and DIBH-TIMAT. Compared with cVMAT-DIBH which contributed significantly higher low dose region of right lung and mean dose of right breast than tangential intensity-modulated radiotherapy-DIBH, DIBH-TVMAT provided better right lung and right breast protection. However, DIBH-TVMAT still had higher V5Gy of right breast than DIBH-TIMRT significantly. Risk of secondary malignancy of right breast should be mentioned and discussed especially with young patients. The limited degree of TVMAT partial arcs we designed not only to provide better dose adjustment planning parameters and similar OAR sparing benefits to DIBH-TIMRT, but also to spare the patients from the disadvantage of low-dose scattering from cVMAT.

Most studies focusing on DIBH for left breast cancer radiotherapy point out the benefit of mean heart dose reduction [5–7]. Treatment plans for breast cancer with DIBH were mainly based on three-dimensional conformal therapy and TIMRT techniques. In Hayden et al.’s study [5], using Hybrid IMRT technique with DIBH resulted in a significant reduction of radiation dosage to the heart and left anterior descending coronary artery compared with FB technique. In our study, DIBH-TVMAT had significantly lower dose of the heart, including mean dosage, D2%, V30Gy, V10Gy and V5Gy than DIBH-TIMAT. For LAD, DIBH-TVMAT had significantly lower Dmax and D10% compared with FB-TVMAT. Similar differences in heart doses for free breathing and moderate deep inspiration breath hold (mDIBH) plans were observed by Comsa et al. [20]. Our results also revealed statistically significant differences of left lung mean dose, D2%, V30Gy, V20Gy, V10Gy and V5Gy between FB and DIBH techniques (all dose parameters *p* < 0.001). The increased low dose region of left lung caused by VMAT-based technique was significantly reduced by DIBH technique. The mean dose reduction of OAR including the heart, left lung, right lung, and right breast from FB-TVMAT to DIBH-TVMAT showed a strong correlation with OAR mean dosages of FB-TVMAT in our study. The strong correlation suggests that the higher the incidental exposure of the OARs with FB-TVMAT technique, the more protection to the organ DIBH-TVMAT can provide. DIBH was revealed to have advantages when combined with IMRT and hybrid IMRT techniques for breast cancer treatment in previous studies, and our study proved that DIBH also has a strong advantage for heart, right lung, and left lung protection when combined with TVMAT technique. For a patient receiving treatment with DIBH technique, several deep-inspiration breathing holds will need to be taken to complete the treatment. The reproducibility would not be perfect for every breathing hold and the perfection of each breathing hold decreases along with the increase of treatment time [21, 22]. More instability leads to more unexpected radiation exposure to organs we are concerned with. A treatment technique which can provide shorter total treatment time can help the patient adapt to the whole treatment course well and improve treatment quality. DIBH-TVMAT can provide not only the advantage from DIBH to increase heart and lung and other OAR sparing, but also to obtain lower delivered MU and shorter total treatment time to help the patient proceed through treatment smoothly.

Only whole breast irradiation plans were included in this study. However, breast tumor bed boost will also be included in real treatment course. The variety of breast tumor locations leads to different boost dose contribution to target left breast, and normal organ such as lung, heart, and contralateral breast. Our patient number is small. The patient number for each breast tumor location is even smaller. For statically comparison, more patient number is needed to get a reasonable analysis of association between tumor locations and dose distribution. Breast tumor bed boost is an important issue in breast radiotherapy. Further study with more patient number is needed for more investigation.

For each patient, DIBH-TVMAT was planned first. The aim for FB-TVMAT plan was to decrease OAR dose as low as possible under the precondition of V95% of PTV ≥ 95%. The chest wall shape of FB-TVMAT was different from DIBH-TVMAT. Under the prerequisite use of same gantry setting and similar V95% of PTV as DIBH-TVMAT but with different chest wall shape, it was hard to achieve similar normal organ protection on FB-TVMAT plans. Our dosimetric data showed lower CI and V95% of PTV of DIBH-TIMRT plans compared with DIBH-TVMAT plans. The average of V95% of PTV of DIBH-TIMRT was 95.8%. The dose of left lung of DIBH-TIMRT might be improved if V95% of PTV continuously decreased, and which would lead to sacrifice of PTV treatment quality.

Better OAR protection might be achieved if gantry setting was changed, however, it would lead to more uncertain factors. Further studies investigating gantry setting change for one patient with different chest wall shape under different condition may be needed.

## Conclusion

DIBH-TVMAT for left breast cancer treatment retains similar treatment plan quality compared to the DIBH-IMRT technique without compromising mean dose and low dose as V10Gy restrictions to the heart, left lung, right breast and right lung. Left lung protection can be increased with the DIBH-TVMAT technique. However, DIBH-TVMAT lead to higher V5Gy to right breast and substantially higher V5Gy to heart. Shorter treatment time provided by DIBH-TVMAT helps patients to receive an entire DIBH treatment course more smoothly. For left breast cancer patients receiving treatment with the DIBH technique, DIBH-TVMAT provides better treatment quality and is an efficient treatment strategy to consider.

## Abbreviations

CI: Conformity index
CT: Computed tomography
CTV: Clinical target volume
cVMAT: Continuous VMAT
D10%: Dose received by 10% of volume
D2%: Dose received by 2% of volume
DIBH: Deep inspiration breathing hold technique
FB: Free-breathing
HI: Homogeneity index
IMRT: Intensity modulated radiation therapy
LAD: Left anterior descending artery
OAR: Organ at risk
PTV: Planning target volume
TIMRT: Tangent-based intensity modulated radiation therapy
TVMAT: Tangent-based volumetric modulated arc therapy
V10Gy: Volume receiving 10Gy or more
V20Gy: Volume receiving 20Gy or more
V30Gy: Volume receiving 30Gy or more
V5Gy: Volume receiving 5Gy or more
V95%: Volume receiving 95% of prescribed dose or more
VMAT: Volumetric modulated arc therapy
